# The Role of Semaphorins in the Pathogenesis of Rheumatoid Arthritis

**DOI:** 10.3390/cells13070618

**Published:** 2024-04-02

**Authors:** Jakub Rosik, Joanna Kulpa, Marcin Szczepanik, Andrzej Pawlik

**Affiliations:** Department of Physiology, Pomeranian Medical University, 70-111 Szczecin, Poland; jakubrosikjr@gmail.com (J.R.); joanna.h.kulpa@gmail.com (J.K.); marcin.t.szczepanik@gmail.com (M.S.)

**Keywords:** semaphorins, angiogenesis, rheumatoid arthritis

## Abstract

Rheumatoid arthritis (RA) is one of the most common autoimmune diseases. Inflammation of the synovial fluid propagates the pathological process of angiogenesis. Semaphorins play a crucial role in the context of endothelial cell function, and their pleiotropic nature has various effects on the further development of RA. This narrative review summarises the various roles of semaphorins in the pathology of RA and whether they could play a role in developing novel RA treatment options.

## 1. Introduction

Rheumatoid arthritis (RA) is a chronic, progressive systemic autoimmune disease. It is one of the most common autoimmune diseases, affecting up to 2% of the global population [1]. The differentiating factors in RA include gender, with a higher prevalence in women, age, and geographical location [2,3]. Large-scale genome-wide association studies (GWASs) have enabled the detection of specific risk genes associated with proinflammatory cytokines, chemokines, signal transducers, transcriptional activators, and pathways, including over 100 single nucleotide polymorphisms (SNPs) located outside of the human leukocyte antigen (HLA) system [4]. RA is a chronic inflammatory connective tissue disease with non-specific synovitis and destruction of joints, periarticular areas, and bones. In addition to joint lesions, the hallmarks of the disease are numerous extra-articular manifestations and systemic complications. All this leads to functional impairment, severe disability, and premature death of patients. The common symptoms reported by patients with RA include joint stiffness usually presenting in the morning, fatigue, joint tenderness, rheumatoid nodules underneath the skin surface, fever, and symmetric inflammation of small joints. The inflammation starts at smaller joints. Over time, tendons and ligaments are weakened [5,6]. RA can be divided into two subcategories: seropositive and seronegative. This division is based on the presence of rheumatoid factor (RF), anti-citrullinated protein antibodies (ACPA), and anti-carbamylated protein antibodies (anti-CarP) [7,8].

Despite several decades of extensive research into the condition, the aetiopathogenesis of RA is still not fully understood [9,10]. The accumulated data has shown that environmental factors, including bacterial and viral infections as well as immune disorders and genetic predisposition, may contribute to the onset of chronic inflammation [6,10,11]. Inflammation is a defensive reaction that can have beneficial effects on the body, including fighting an infection, but the inflammatory response can sometimes be pathologically prolonged or inappropriate to the triggering stimulus. If the inflammatory response is prolonged, then chronic inflammation develops, which is the main characteristic of RA [12,13]. Various types of endogenous mediators that are triggered at the site of the reaction are responsible for the formation and overall course of the inflammatory response. Such mediators include cytokines, a group of low-molecular-weight proteins that control and influence all stages of the immune response [7,9].

The immune response consists of three phases: the induction phase, the effector phase, and the quenching phase. Each phase is tightly controlled by a network of cytokines that regulate each other [9]. Physiologically, the activation and deactivation of individual cytokines occurs at precise times, necessary only for the elimination of the invading agent and its metabolites [13]. Once the damage is repaired, thanks to immunoregulatory mechanisms, the process is inhibited and then extinguished. However, when, over a prolonged period of time, the balance between mediators capable of eliciting an immune response and their inhibitors is disrupted, the chronic immune-inflammatory state observed in RA can occur [7,12].

RA is an autoimmune disease, which means that the basis for its development is defective immunoregulation of the body. The complexity of RA pathogenesis lies in the involvement of cells of the innate and acquired immune system; they function in an abnormal manner, leading to defective immunoregulation that is the cause of the initiation and maintenance of RA [7,13]. The beginning of the inflammatory process in the synovial membrane of the joints is considered to be the presentation of an as-yet-unidentified antigen to helper T cells by antigen presenting cells (APCs). Helper T cells activate CD4+ T lymphocytes, which initiate the effector phase of the immune response. T cells are responsible for several other destructive processes leading to the development of RA [6]. In the synovial membrane of the joint, activated T cells are capable of destroying periarticular tissues by continuously sustaining inflammation through stimulation of the synthesis of a number of pro-inflammatory cytokines, including tumour necrosis factor alpha (TNFα), interleukin 1 (IL-1), IL-2, interferon γ (INF-γ), matrix metalloproteinases (MMPs), and other molecules [6,9,14]. In addition, T cells initiate the proliferation of B cells, synoviocytes, and fibroblasts, resulting in increased antibody secretion. B cells that differentiate into plasmocytes begin synthesising autoantibodies to molecules including cyclic citrullinated peptide (ACPA) and RF, which are used in the diagnosis of RA [6]. Other cell types, such as macrophages, neutrophils, and mast cells, among others, contribute to RA pathogenesis by sustaining chronic inflammation [6]. In the context of immune dysfunction in RA, the crucial role of humoral factors cannot be overlooked. These pro-inflammatory cytokines—TNFα, IL-1β, IL-6, IL-8, IL-17, IL-21, and IL-23—stimulate numerous processes that lead to cartilage degradation [6,9,14].

Reducing regulatory T cell (Treg) function plays a crucial role in the pathogenesis of autoimmune diseases, including RA. Moreover, research about Tregs might shed light on the therapeutic potential of recovering Treg function. Such an approach might enable the re-establishment of self-tolerance [15].

Currently, new mediators are being sought to influence the development and course of RA. Studies in recent years have revealed a potential role for semaphorins in the pathogenesis of RA. This review analyses the role of semaphorins in the progression of RA, specifically, their role in the pathological angiogenesis.

## 2. Semaphorins

Semaphorins are a group of 30 known glycoproteins that are essential for cellular development, maintenance, and functioning. Their division is based on protein domain structures as well as the species of origin and structural motifs. There are eight classes of semaphorins, of which, classes 3–7 are found in vertebrates [16,17] and participate in the development of the cardiovascular system and the pathological processes of autoimmune diseases [18,19]. Semaphorins act as signalling ligands, regulating the morphology and motility of cells that comprise the cardiovascular and immune systems. Moreover, they play a vital role in the development of the nervous system through the regulation of neuronal proliferation, polarity, synapse formation, and function [16,20]. They also act as cues for axons and dendrites [16].

**Figure 1:**
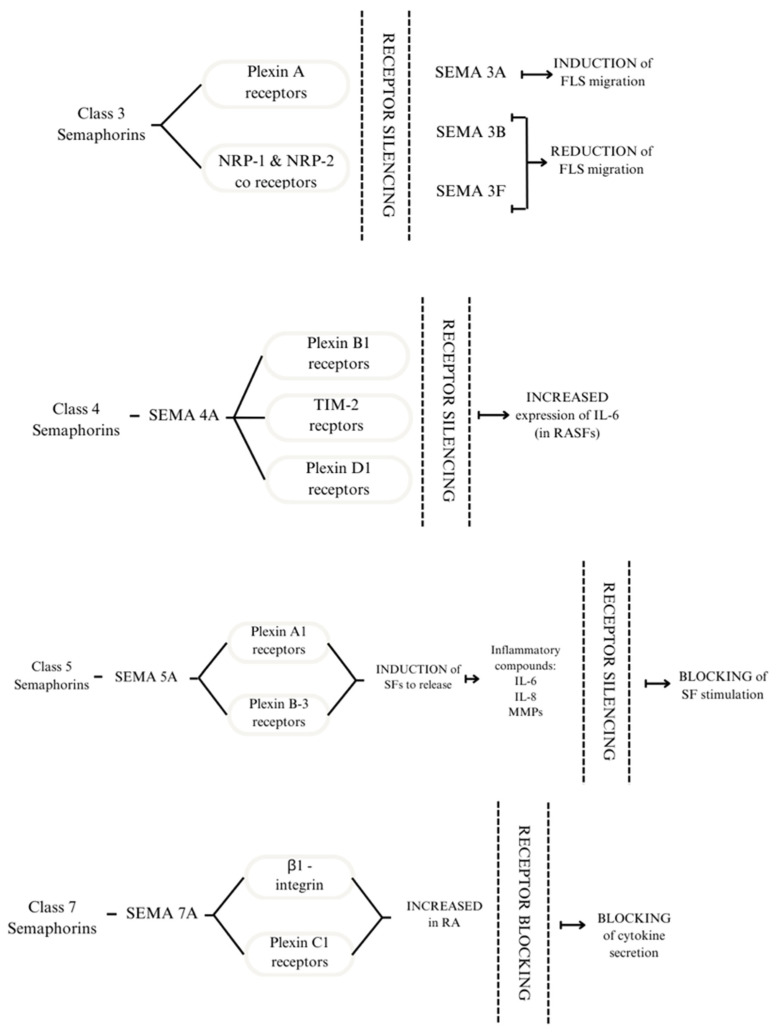
Receptors to which Class 3 semaphorins bind are plexin A receptors and NRP-1 and NRP-2 co-receptors. Silencing the receptors modulates the response of FLS. SEMA4A binds to plexin B1, TIM 2, and D1 receptors. Silencing the receptors above leads to increased expression of IL-6 in RASFs. SEMA5A interacts with plexin A1 and plexin B3; this process induces SFs to release inflammatory compounds: IL-6, IL-8, and MMPs. By silencing the receptors, SF stimulation is blocked. The effect of SEMA7A is mediated through β1-integrin and plexin C1. These receptors are increased in patients with RA. By blocking them, the cytokine secretion is also blocked [21,22,23,24].

Semaphorin signalling is transduced through neuropilins and plexins, transmembrane proteins that act as receptors (Figure 1). The interaction between them occurs due to the dimerisation of extracellular domains, thus forming complexes capable of transducing signals [18]. The role of semaphorins in RA can be explored through intracellular signal transduction pathways. One study using plexin C1 and β1-integrin as SEMA7A receptors focused on its effect on T cells and monocytes. The results showed an increase in plexin C1 and β1-integrin in patients with RA compared with healthy subjects [21].

Semaphorins play a crucial role in the functioning of immune cells that invade the synovial tissue. They are produced by endothelial cells, T cells (Tregs, activated CD4+ and CD8+), regulatory B cells (Bregs), macrophages, and DCs [25]. Upon arrival, activated T cells and macrophages release proangiogenic cytokines, namely IL-6, TNF-α, and IL-17 [21]. Cytokines involved in the pathogenesis of RA are IL-6, granulocyte–macrophage colony-stimulating factor (GM-CSF), and IFNs. Their significance is connected to the activation of rheumatoid arthritis synovial fibroblasts (RASFs) and the inflammatory milieu [26]. Semaphorins can be expressed by immune cells and thus modulate immune responses. This process is a critical element of the pathophysiology of autoimmune diseases such as RA [18].

**Figure 2:**
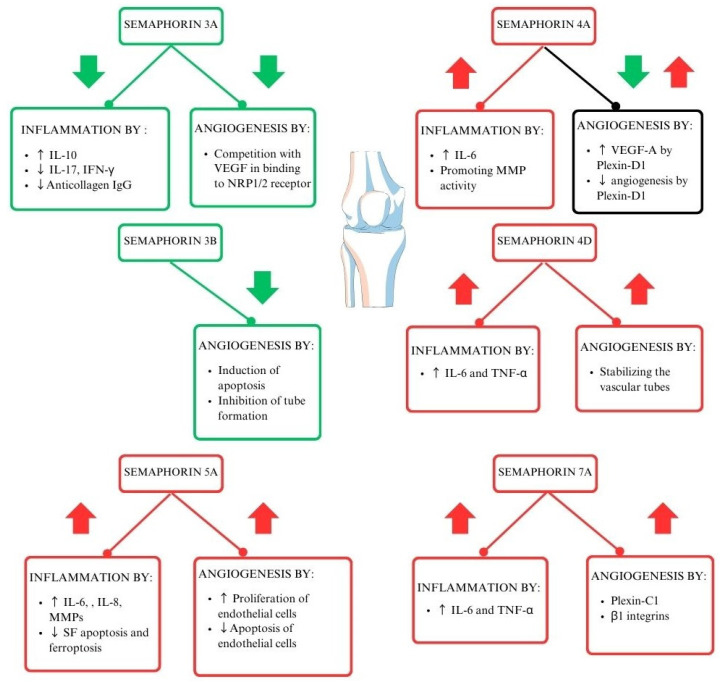
The role of semaphorins in the pathogenesis of RA (29–33). Interleukin 6 (IL-6), interleukin 8 (IL-8), tumor necrosis factor–alpha (TNF-α), vascular endothelial growth factor (VEGF), interferon-gamma (IFN-γ), neuropilin-1/2 (NRP-1/2), metalloproteinases (MMPs), interleukin 17 (IL-17), synovial fibroblasts (SFs).

Each of the semaphorin classes works in its unique way (Figure 2) [18]. Class 3 semaphorins are considered immunoregulators in autoimmune diseases. It is worth noting that SEMA3A regulates the function of T lymphocytes and plays a crucial role in decreasing inflammation in RA. SEMA3A induces the production of the anti-inflammatory IL-10 by T lymphocytes with NP-1 receptors. Moreover, several other proinflammatory cytokines are downregulated by Sema3A, such as IL-17 and IFN-Ɣ [27]. Class 4 semaphorins act as immunostimulators. Their involvement in RA activity is correlated to elevated levels of swollen joint markers [23,28]. Another immunostimulator and representative of class 5 semaphorins is SEMA5A. Its role is connected to increased secretion of proinflammatory factors and lowering crucial cell functions such as programmed cell death [29]. Moreover, the immunostimulating function can be attributed to SEMA7A, from class 7 semaphorins. It enhances cytokines contributing to RA progression, such as TNF-α and IL-6, IFN-γ, IL-17, IL-22, and Th17/Tc17 [21,30,31,32,33]. The role of semaphorins in the pathogenesis of RA is shown in Figure 2.

## 3. Angiogenesis and Destruction of the Synovial Joint

Angiogenesis plays a key role in the early stages of RA, especially in the proliferation of synovial tissue and the formation of pannus [34]. Semaphorins can stimulate and inhibit angiogenesis. Their role in angiogenesis was scrutinized in various cancers [35].

RASFs invade and destroy cartilage and bone during inflammation. Physiologically, synovial fibroblasts (SFs) provide nutrients to the joints and also take part in joint remodelling [36]. Another participant in the process, synovial stromal fibroblast-like synoviocytes (FLSs), are responsible for the release of MMPs, aggrecanases, and other proinflammatory cytokines, which destroy cartilage and extracellular matrix. They show resistance to apoptosis and promote the expression of receptor activator for nuclear factor κ B ligand (RANKL) [22]. Inflammation promotes angiogenesis, which may enhance tissue inflammation and contribute to tissue growth. Inhibiting angiogenesis could reduce inflammation and ameliorate the progression of RA in the synovial tissue [37]. Several semaphorins play a vital role through their cues; some promote angiogenesis and others inhibit the process [18,19,31].

There are two theories that best explain the role of angiogenesis regarding inflammation in RA. According to the first one, secreted mediators and inflammatory cells infiltrating tissue are induced prior to angiogenesis. This theory suggests that inflammation stimulates angiogenesis due to hypoxia, which upregulates the release of angiogenic growth factors, for example, vascular endothelial growth factor (VEGF) [28,37]. VEGF also regulates bone growth. Regarding RA, VEGF is released in synovial fluid and patients with RA have a higher VEGF concentration than patients with osteoarthritis (OA). It predominantly occurs in synovial lining cells in joints. VEGF mediates angiogenesis and endovascular permeability in new tissues [38]. A more recent study has suggested that angiogenesis precedes the infiltration of leukocytes (tissue inflammatory cells). Already existing vessels allow leukocytes to enter the synovial sublining layer, promoting or intensifying inflammation. Subsequently, VEGF binds to its receptor on endothelial cells and stimulates those cells to release proteolytic enzymes. MMPs degrade other parts of joints, such as the basement membrane. This process enables further endothelial proliferation [39]. Of note, this second theory is based on experimental models of inflammatory diseases.

SEMA3 is a vital regulator of angiogenesis [40]. SEMA3A injection in a mouse model of asthma prevented infiltration of the bronchioles and arterioles by leukocytes. Moreover, SEMA3A-treated mice had reduced concentrations of micro-vessels. It is possible that SEMA3A potential in reducing angiogenesis might be transferred to an RA model [41]. SEMA3E decreased angiogenesis markers, like VEGF, in an animal model [42]. Most likely, SEMA3 might contribute to endothelial cell apoptosis [40]. Moreover, due to binding to the receptor plexinD1, SEMA3E inhibits endothelial cell adhesion, and SEMA3 F, interacting with plexinA1, inhibits the migration of endothelial cells. However, occasionally, SEMA3 may also promote angiogenesis [40]. Serum levels of SEMA4A and SEMA4D are correlated with angiogenesis markers like VEGF or TIE-2 [28]. SEMA7A promotes angiogenesis through various mechanisms [18]. Further investigation of the mechanisms of semaphorins in angiogenesis may contribute to novel therapeutic agents in autoimmune diseases.

## 4. The Possible Use of Semaphorins in RA Treatments

The current pharmacological treatments for RA include Janus kinase (JAK) inhibitors, such as baricitinib and tofacitinib; disease-modifying antirheumatic drugs (DMARDs), such as methotrexate (MTX) and leflunomide; and anti-cytokine drugs, such as adalimumab and infliximab [26,43]. The most commonly used drug, and very often the first choice, is MTX. The next step can be adding sulfasalazine or leflunomide [44,45]. The possible use of semaphorins as a treatment target for patients with RA has yet to be established due to the early nature of research regarding their usefulness [46] (Figure 1).

### 4.1. Class 3 Semaphorins and RA

Class 3 semaphorins were initially considered to compete with VEGF and thus inhibit VEGF-induced angiogenesis [47], but they are now known to be involved in pathological angiogenesis and vessel remodelling rather than developmental angiogenesis (Figure 2). Expression of class 3 semaphorins is reduced in the synovial tissue of patients with RA [48]. Importantly, HOXA5, a transcription factor crucial for class 3 semaphorin expression, is also significantly reduced in the synovium of patients with RA [48].

Analysis of baseline messenger RNA (mRNA) expression of class 3 semaphorins showed a significant reduction in SEMA3B and SEMA3F in patients with RA compared with patients with undifferentiated arthritis [22]. A follow-up study showed further downregulation of SEMA3B, SEMA3C, SEMA3F, SEMA3G, and neuropilin-1 (NRP-1) mRNA expression at baseline in patients with persistent RA compared with patients with self-limiting disease [22].

**Figure 3:**
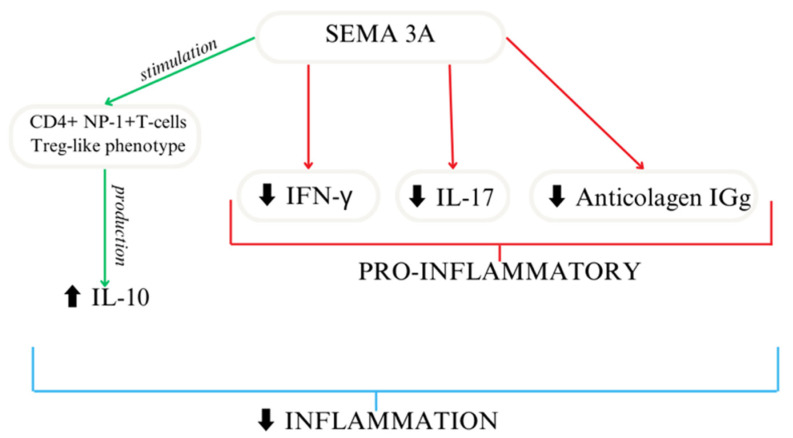
SEMA3A importance as an immunoregulator [27].

Based on the available research, SEMA3A seems to act as an immunoregulator [49,50] (Figure 3). In another study, researchers reported similar results using collagen-induced mice to mimic arthritis, where treatment focused on the overexpression of SEMA3A led to decreased anti-collagen IgG and the suppression of IFN-γ and IL-17. SEMA3A treatment significantly reduces renal damage in a lupus nephritis mouse model. Moreover, it delays the onset of proteinuria and improves survival [51]. Moreover, SEMA3A increased the differentiation of macrophages as well as T cells [27]. A shorter SEMA3A derivative, called truncated SEMA3A, induces the expression of regulatory cytokines like IL-10 and TGF-β and reduces the secretion of pro-inflammatory ones. It can be transfected using a viral vector. Its function in preserving immune homeostasis makes it a potential therapeutic agent [52].

**Figure 4:**
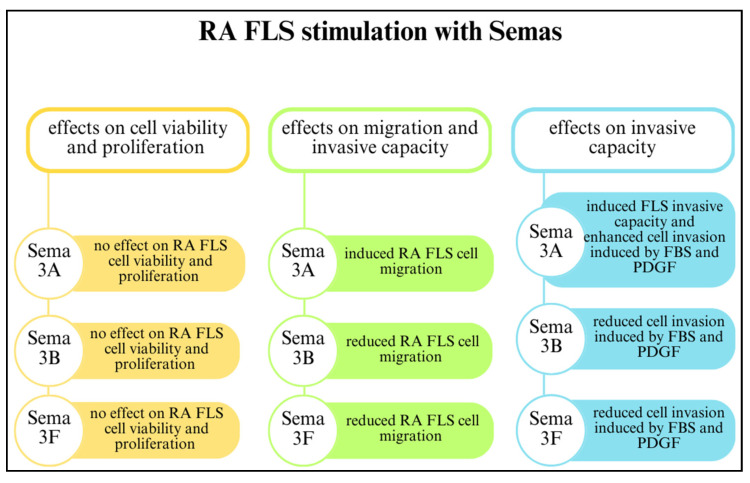
The effects of class 3 semaphorins on rheumatoid arthritis fibroblast-like synoviocytes (RA FLSs) [22,49]. Abbreviations: foetal bovine serum (FBS); platelet-derived growth factor (PDGF).

Class 3 semaphorins participate in FLS-mediated joint destruction and regulate the invasive activity of FLSs. Reducing SEMA3B and SEMA3F expression through initial inflammatory cues in early arthritis may have a crucial impact on disease development by suppressing FLSs. The involvement of SEMA3B and SEMA3F in RA is related to FLS-mediated joint destruction and MMP production. However, class 3 semaphorins do not regulate FLS proliferation and viability. The effects of SEMA3A, SEMA3B, and SEMA3F on RA FLSs are shown in Figure 4 [22].

Several studies using data collected from systemic lupus erythematosus (SLE), RA, OA, and control groups found a significant decrease in SEMA3A in the SLE and RA groups compared with the control and OA groups [49,53,54]. Moreover, there was a negative correlation between the serum SEMA3A level and the erythrocyte sedimentation rate (ESR), C-reactive protein (CRP), and Disease Activity Score 28 (DAS28) [49,54,55]. However, Gao et al. [56] reported contradictory results from a different population.

Studies have shown a decreased expression of SEMA3A in the serum of patients with RA compared to control groups [5,57]. As suggested by Takagawa et al., serum levels found in RA patients may be linked to the quantity and lower expression of SEMA3A in joints [58]. In part, this may be because of SEMA3A’s solubility.

Interestingly, the balance between SEMA3A and VEGF expression could regulate processes connected with RA disease activity, such as inflammation, angiogenesis, and synovial cell proliferation [49]. SEMA3A administration attenuates joint tissue damage via the inhibition of several VEGF-induced processes, including endothelial cell proliferation and migration and FLS invasion [54]. The urine SEMA3A level is also a potent disease activity marker. Its negative correlation with disease occurrence and renal involvement has been reported for SLE [59].

Researchers have reported a negative correlation between SEMA3B, SEMA3F, and SEMA3G levels and parameters for RA disease activity, namely DAS28, the ESR, and CRP levels [22,60]. Class 3 semaphorin expression in synovial tissue of the studied patient group was reduced and negatively correlated with disease activity parameters [22]. Moreover, the protective role of SEMA3B might vary depending on the disease stage [60]. Another significant finding was the reduced SEMA3B, SEMA3C, SEMA3F, and SEMA3G mRNA expression in patients with early arthritis. Furthermore, SEMA3B and SEMA3F protein levels were decreased. Collectively, these findings demonstrate that major cell populations from the inflamed synovial tissues expressed SEMA3B and SEMA3F by FLSs but not by monocytes or macrophages [22]. Nevertheless, a study of a mouse model and 28 patients with RA reached different conclusions [61]. Expression of SEMA3G and its receptor neuropilin-2 on activated macrophages in the synovium exacerbates joint inflammation. SEMA3G is a potent enhancer of bone marrow-derived macrophage proliferation and migration [61].

The mechanism that allows for FLS inhibition by SEMA3B and SEMA3F was speculated to be connected to inhibited activation of extracellular signal-regulated kinase (ERK) and the small GTPase Rac1. ERK was elevated in the synovial tissues of patients with RA compared with the control group. Researchers made similar observations in patients with early arthritis who had developed erosive RA. The results suggest that the inhibitory properties of SEMA3B and SEMA3F regarding the FLS invasive capacity may be linked to molecular pathways with ERK [22].

### 4.2. Class 4 Semaphorins and RA

**Figure 5:**
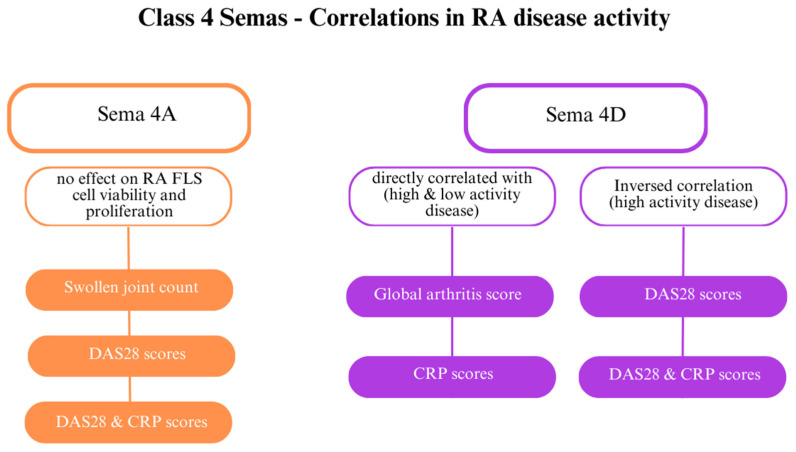
The role of class 4 semaphorins in rheumatoid arthritis (RA) [23]. Abbreviations: C-reactive protein (CRP); Disease Activity Score 28 (DAS28); fibroblast-like synoviocytes (FLSs).

SEMA4D plays several roles in RA; it is involved in angiogenesis and immune activation. While SEMA4D levels are elevated in the serum and synovial fluid of patients with RA, they have an inverse correlation with CRP levels and DAS28 (Figure 5) [28]. Furthermore, ADAMTS-4 (aggrecanase-1), an enzyme that generates a soluble, active form of SEMA4D and causes cartilage degradation, is more concentrated in patients with RA than in healthy individuals. SEMA4D and ADAMTS-4 have been proven to interact. Other indicators directly correlated with SEMA4D are the global arthritis score and CRP levels [32]. Tissue inhibitor of metalloproteinase 3 (TIMP-3) inhibits MMPs, including MMP-13 and ADAMTS-5, which destroy collagen type II [62].

Soluble SEMA4D increases the production of TNF-α and IL-6 by monocytes; the former cytokine increases ADAMTS-4 levels. These two cytokines are known to induce osteoclastogenesis via elevated RANKL production. An anti-SEMA4D antibody downregulated TNF-α and IL-6, reducing pathological angiogenesis and inflammatory infiltration into the synovium [32]. SEMA4D might serve as a predictor for radiographic progression of RA. Ha et al. [63] observed higher SEMA4D concentrations in patients with radiographic progression than in patients without progression.

Researchers have also examined the connection between class 4 semaphorins and other indicators of RA, including DAS28 and CRP levels. Patients with highly active RA (DAS28 > 5.1) and patients with low activity (DAS28 score < 3.2) showed elevated class 4 semaphorin levels. SEMA4A might be involved in RA activity [64]. Elevated SEMA4A levels are directly correlated with DAS28 and DAS28-CRP, markers of the swollen joint count [23,28]. SEMA4A influences the activity of RASFs. Recombinant human SEMA4A (rhSEMA4A) promotes a greater invasive ability of RASFs compared with silencing SEMA4A with small interfering RNA (siRNA), which blocks invasion. Furthermore, rhSSEMA4A induces the expression of MMP-3 and MMP-9, which degrade the extracellular matrix and promote RASF invasion.

SEMA4A might also participate in inflammation by promoting MMP activity and increasing IL-6 production by RA FLSs. The data suggest that SEMA4A and SEMA4D compete for binding to their common receptor, plexin D1. These semaphorins present opposite effects [28]. THP-1 cells, a macrophage cell line, play a role in RA–induced by lipopolysaccharides (LPS) and showed higher SEMA4A expression and, consequently, increased TNF-α and IL-1β secretion [23]. SEMA4A is also correlated with TNFα; stimulation with this cytokine increases the SEMA4A level, but stimulation with IL-6 does not.

When comparing synovial tissue samples from patients with RA and patients with OA, SEMA4A was higher in patients with RA [23]. However, even higher SEMA4A levels are observed in patients with SLE [65]. Soluble and membrane-bound SEMA4A concentrations have been proposed as biomarkers for the differential diagnosis between RA and SLE [65].

In another study on two independent cohorts of 141 patients with RA, a markedly increased SEMA4A level was a predictor of treatment failure (hazard ratio [HR] 2.71, 95% confidence interval [CI] 1.14–6.43). The specificity was even higher when the SEMA4A level was combined with DAS 28-CRP > 3.2 or Doppler ultrasound screening (HR 10.42, 95% CI 1.41–76.94) [66].

Finally, SEMA4G has been subject to research because a SNP near this gene reached genome-wide significance for the certolizumab pegol response [67].

### 4.3. Class 5 Semaphorins and RA

SEMA5A has been elevated in the serum of patients with SLE and RA. Researchers reported a high SEMA5A level in synovial macrophages (SMs) from patients with RA compared with patients with OA [68,69]. SEMA5A has a substantial impact on RA pathogenesis: it facilitates pannus formation [46] and induces the proliferation and activation of T cells and natural killer (NK) cells, which secrete higher amounts of pro-inflammatory factors. SEMA5A attaches to functional receptors such as plexin-A1 and plexin-B3 on inflammatory cells [29]. Plexins are fully functional SEMA5A receptors, and silencing plexin expression blocks SEMA5A-mediated SF stimulation [24]. SEMA5A induces SFs to release inflammatory compounds, including IL-6, IL-8, and MMPs.

Furthermore, SEMA5A decreases SF apoptosis and ferroptosis, two forms of programmed cell death. Ferroptosis is regulated by glutathione peroxidase 4 (GPX4), which stops the production of lipid peroxidation products [29]. A recent study suggests that the overwhelming majority of SEMA5A in the synovial fluid comes from SMs; only a tiny fraction is from SFs [24]. Moreover, other studies confirm that ferroptosis can be reduced by SEMA5A through activation of the phosphoinositide 3-kinase (PI3K)/AKT/mammalian target of the rapamycin (mTOR) pathway, leading to higher levels of GPX4. This pathway might also promote the activation of SFs. Therefore, SEMA5A increases SREBP1/SCD-1 signalling activity, suppressing ferroptosis [24].

Research also indicates that SEMA5A plays a significant role in angiogenesis. In one study, researchers generated three SEMA5A constructs: one without any changes, one without the TSP-1 domain, and one without PSI-TMR. There was a marked decrease in angiogenesis for SEMA5A lacking the TSP1 domain compared with intact SEMA5A. Furthermore, the authors found that SEMA5A lacking the TSP1 domain decreased the levels of inflammatory agents and pannus damage and increased IL-10, an anti-inflammatory cytokine. These findings suggest that TSP1 is a key domain in the pathological function of SEMA5A in RA [70].

Researchers have reported that SEMA5A increases the levels of T cells and NK cells. While SEMA5A stimulation alone only modestly increased the levels of these cells, the addition of IL-2 and IL-15 alongside SEMA5A enhances this response [71].

Qin et al. [46] developed a human antibody SYD12-12 blocking SEMA5A to evaluate its efficiency in a collagen-induced arthritis (CIA) mouse model. Treatment stopped disease progression, denoted by inhibition of angiogenesis, synovial hyperplasia, pannus formation, and bone destruction, thus proving the therapeutic potential of blocking SEMA5A.

### 4.4. Class 7 Semaphorins and RA

Similarly to SEMA4D, SEMA7A acts as one of the mediators in pathways important for RA development. Based on observation in an experimental model of autoimmune arthritis, blocking SEMA7A-β1 signalling significantly reduces RA symptoms [72]. SEMA7A participates in the primary immune response by regulating interactions between T cells and monocytes. Further analysis revealed that soluble SEMA7A regulates cytokine secretion by T cells and monocytes. The observed cytokine production indicates that stimulation with SEMA7A enhances the production of TNF-α and IL-6, cytokines that are known to play a role in RA progression [21,30,33].

SEMA7A could represent a therapeutic target to counteract RA progression. In a study focused on the potential therapeutic use of SEMA7A in RA, the authors compared soluble SEMA7A levels in serum and synovial fluid from patients with RA and patients with OA [21]. The comparison was based on the cell surface and SEMA7A transcript levels in T cells and monocytes. The authors identified a significant correlation between key indicators of RA activity and progression, including DAS28, CRP, and RF levels, and serum soluble SEMA7A [30]. As previously stated, there have been similar conclusions from studies that analysed the roles of other semaphorins. Furthermore, research from our group suggests that soluble SEMA7A is a potentially helpful marker in RA progression [21].

The evaluation of SEMA7A has also focused on the increased production of the cytokines IFN-γ, IL-17, IL-22, and Th17/Tc17 [21]. Moreover, stimulation using SEMA7A upregulated the expression of transcription factor T-bet and RORγt in CD4+ T cells [73]. However, upon further analysis, SEMA7A-induced cytokine secretion was only attenuated by the blockage of β1-integrin. The authors concluded that β1-integrin in monocytes leads to focal adhesion kinase (FAK) activation [74,75,76,77,78].

## 5. Conclusions

RA is a chronic autoimmune disease in which inflammation develops in the tissues that make up the joints. Abnormal interactions between cells, inflammatory mediators, antibodies, and signal transduction pathways lead to abnormal activation of the immune system and, consequently, the development of clinical changes. During the development of the disease, the synovial membrane is infiltrated by B cells, T cells, and monocytes, causing neo-angiogenesis and activation of endothelial cells and synoviocytes. Through synovial membrane proliferation and the secretion of inflammatory mediators by synoviocytes, articular cartilage is damaged and bone resorption is increased. Due to the progressive course of the disease leading to patients’ disability, a number of mediators are currently being sought that may be involved in the pathogenesis of this disease and may be markers to help monitor its course, as well as potential targets for therapy. Studies in recent years have demonstrated the important role of semaphorins in the development of RA. They exert multiple effects and influence immune response cells, the synthesis of pro-inflammatory mediators, and angiogenesis to modulate contribute to RA pathogenesis. Class 3–7 semaphorins seem to be important compounds regulating the process of inflammation in RA and may also be new targets for RA therapy. However, this eventuality requires further research. The animal model results are promising and suggest that further research may lead to the development of new treatment options for patients with RA in the future. However, a thorough understanding of the role of semaphorins in the pathogenesis of RA requires many clinical studies.
